# A decreased level of high‐density lipoprotein is a possible risk factor for type 2 diabetes mellitus: A review

**DOI:** 10.1002/hsr2.1779

**Published:** 2023-12-20

**Authors:** Ali Bayat Bodaghi, Erfan Ebadi, Mohammad Javad Gholami, Reza Azizi, Aref Shariati

**Affiliations:** ^1^ Student Research Committee Khomein University of Medical Sciences Khomein Iran; ^2^ Molecular and Medicine Research Centre Khomein University of Medical Sciences Khomein Iran

**Keywords:** apo A‐I, diabetes, high‐density lipoproteins, prevention

## Abstract

**Introduction:**

Type 2 diabetes mellitus (T2DM) is characterized primarily by dyslipidemia and hyperglycemia due to insulin resistance. High‐density lipoprotein (HDL) play a significant role in preventing the incidence of dyslipidemia and its complications. HDL has different protective functions, such as reducing oxidation, vascular inflammation, and thrombosis; additionally, its anti‐diabetic role is one of the most significant recent discoveries about HDL and some of its constituent lipoproteins.

**Methods:**

This research reviews ongoing studies and preliminary investigations into the assessment of relation between decreased level of HDL and T2DM.

**Results:**

The levels of HDL and its functions contribute to glucose hemostasis and the development of T2DM through four possible mechanisms, including insulin secretion by beta cells, peripheral insulin sensitivity, non‐insulin‐dependent glucose uptake, and adipose tissue metabolic activity. Additionally, the anti‐oxidant properties of HDL protect beta cells from apoptosis caused by oxidative stress and inflammation induced by low‐density lipoprotein, which facilitate insulin secretion.

**Conclusion:**

Therefore, HDL and its compositions, especially Apo A‐I, play an important role in regulating glucose metabolism, and decreased levels of HDL can be considered a risk factor for DM. Different factors, such as hypoalphalipoproteinemia that manifests as a consequence of genetic factors, such as Apo A‐I deficiency, as well as secondary causes arising from lifestyle choices and underlying medical conditions that decrease the level of HDL, could be associated with DM. Moreover, intricate connections between HDL and diabetic complications extend beyond glucose metabolism to encompass complications like cardiovascular disease and kidney disease. Therefore, the exact interactions between HDL level and DM should be evaluated in future studies.

## 1 INTRODUCTION

Approximately 150 million people worldwide suffer from diabetes mellitus (DM), a common metabolic disease that is characterized primarily by dyslipidemia and hyperglycemia due to insulin resistance (IR) that could enhance the chance of different disorders and infections.^1^, ^2^, ^3^, ^4^ To this end, in patients with type 2 diabetes mellitus (T2DM), the uptake of glucose from the blood into the tissues is impaired. Additionally, impaired lipid, protein, and carbohydrate metabolism with a combination of hyperglycemia and dyslipidemia is common in diabetic patients.^1^, ^3^, ^5^, ^6^, ^7^

Dyslipidemia in diabetics is characterized by an increase in triglycerides (TGs) and a reduction in high‐density lipoproteins (HDL).^8^ This type of dyslipidemia is primarily caused by impaired insulin sensitivity in the liver and adipose tissue.^9^ Therefore, there is an increase in triglyceride‐rich lipoproteins, which is commonly associated with decreased HDL levels and increased low‐density lipoproteins (LDLs), which consequently results in hypertriglyceridemia.^10^ It is also demonstrated that cholesterol hemostasis is necessary for insulin secretion from pancreatic beta cells.^5^

HDL, which is mostly known as “good cholesterol,” plays a significant role in preventing the incidence of dyslipidemia and its complications.^9^, ^11^ HDL consists of several components, including apolipoproteins, enzymes, and lipids. Among its constituent apolipoproteins or apoproteins, Apo lipoprotein (Apo) A‐I is the main protein which regulates blood levels of HDL‐C.^9^, ^12^ Several protective functions of HDL, such as reducing oxidation, vascular inflammation, and thrombosis, improving endothelial function, enhancing insulin sensitivity, antiatherogenic function, and angiogenesis, have been identified.^13^, ^14^ Furthermore, its antidiabetic role is one of the most significant recent discoveries about HDL and some of its constituent lipoproteins. Furthermore, it has been indicated that HDL is likewise a significant component in the survival of pancreatic beta cells and inhibits beta cell apoptosis.^3^, ^14^

Diabetes and dyslipidemia have become more prevalent over the past few years and influenced the quality of human life.^9^ Recent studies have shown that HDL‐C is inversely correlated with the incidence of diabetes.^15^, ^16^ In fact, decreased levels of HDL‐C could increase the risk of T2DM in different ethnicities and age groups.^15^ Therefore, in the present review, we discussed different factors that lead to a decreased level of HDL and its possible role in the development of DM.

## 2 HDL‐C: THE GOOD CHOLESTEROL

HDL‐C, also known as “Good cholesterol,” is responsible for carrying 25%–30% of the lipids in the blood circulation.^17^, ^18^ HDL is the smallest lipoprotein in the blood and consists of heterogeneous particles of diverse sizes, charges, shapes, and densities.^19^, ^20^ Proteins and lipids are the most abundant contents of HDL.^20^ Several factors, such as Apo A‐I, ATP‐binding cassette transporter A1 (ABCA1), ATP‐binding cassette subfamily G member 1 (ABCG1), and lecithin‐cholesterol acyltransferase (LCAT) enzyme, are involved in HDL formation and heterogeneity.^19^, ^21^ The HDL lipoprotein contains a lipid core and a protein surface, with Apo A‐I being its dominant protein.^22^ Noteworthy, Apo A‐I accounts for 70% of HDL proteins, which are secreted from the liver (80%) and intestine (20%) and are necessary for HDL assembly.^21^, ^22^ Moreover, Apo A‐I participates in reverse cholesterol transport (RCT) and mediates cholesterol efflux from peripheral tissues.^21^, ^22^

HDL is divided into different sub‐fractions based on their different physiochemical properties.^17^ Based on two‐dimensional gel electrophoresis (2‐DE) and nuclear magnetic resonance spectroscopy, recent studies have found that HDL come in several different types.^18^ To this end, HDL subclasses are HDL_3c_, HDL_3b_, HDL_3a_, HDL_2a_, and HDL_2b_.^18^, ^23^ Distinguishing between the subclasses of HDL can contribute to the success of statin therapy by influencing the function and metabolism of HDL.^17^

HDL has antithrombotic function, improves endothelial repair, inactivates toxic substances by binding to them, and modulates immune cells inflammatory responses.^20^ HDL mediates RCT by taking cholesterol from peripheral cells and returning it to the liver for further metabolism and excretion.^19^, ^24^, ^25^ Cholesterol ester transfer protein (CETP) is one of the major enzymes involved in RCT.^5^ This enzyme is a key component of the pathophysiology of IR in T2DM.^5^ Collectively, HDL has many beneficial roles, but RCT is one of the most important.^9^ It is further indicated that HDL improves glycemic control and has antidiabetic functions; this aspect of HDL function and its mechanisms will be discussed completely in the next parts.^18^, ^19^

## 3 HDL AND GLUCOSE METABOLISM

Diabetic dyslipidemia is characterized by increased triglyceride levels and decreased HDL‐C.^2^, ^26^ It has been demonstrated that HDL‐C is associated with T2DM risk and directly influences glucose metabolism.^27^ The levels of HDL and its functions contribute to glucose hemostasis and the development of T2DM through four possible mechanisms, including: insulin secretion by beta cells, peripheral insulin sensitivity, non‐insulin‐dependent glucose uptake, and adipose tissue metabolic activity.^9^ There is also evidence that HDL not only has antidiabetic properties but also improves IR by facilitating the release of insulin from beta cells.^28^ A decreased level of HDL_2_ particles has been reported in T2DM patients; therefore, the HDL_2_ sub‐fractions are inversely related to glycemic control and the incidence of T2DM.^27^, ^28^

Triglyceride‐enriched particles that accelerate the progression of diabetes are observed in T2DM patients with lower levels of Apo A‐I.^29^ It has also been demonstrated that reconstituted high‐density lipoprotein (rHDL) infusions, which are synthesized HDL drug delivery platforms that possess many of the same advantages as HDL and lead to increased HDL‐C and Apo A‐I levels, enhance glycemic control by increasing RCT and antioxidant capacity.^30^, ^31^, ^32^ The antioxidant properties of HDL protect beta cells from apoptosis caused by oxidative stress and inflammation induced by LDL, which facilitate insulin secretion.^33^

In vivo studies have demonstrated that HDL and its compositions, especially Apo A‐I, play an important role in regulating glucose metabolism.^25^ According to such studies, HDL can have antidiabetic functions, which is a significant advance in the diagnosis, treatment, and management of diabetic patients.

## 4 THE POSSIBLE CORRELATION BETWEEN HDL AND IR

IR is a metabolic disorder characterized by the impaired response of cells to insulin. It plays a pivotal role in the development and progression of various metabolic conditions, including T2DM. IR has a significant impact on lipid metabolism, particularly the alteration of plasma HDL levels.^34^ Low HDL levels frequently accompany IR, contributing to a heightened risk of cardiovascular complications.^35^ Here we aim to explore the intricate relationship between IR and low HDL levels in diabetic patients, providing insights into the underlying pathophysiological mechanisms and potential clinical ramifications.

Firstly, it is essential to understand the fundamental aspects of IR. Insulin, produced by the pancreas, regulates glucose metabolism, promoting its uptake into cells, primarily in muscle and adipose tissue, and also promoting its storage as glycogen or fat. In individuals with IR, cells exhibit reduced sensitivity to insulin, resulting in diminished glucose uptake and elevated blood glucose levels. As this state of IR progresses further, pancreatic beta cells compensate by producing more insulin, leading to a state called hyperinsulinemia. This dysregulation of insulin signaling pathways disrupts the delicate balance of lipid metabolism, contributing to abnormalities in lipid profiles and consequently leading to the dyslipidemia observed in IR individuals. Among the alterations in lipid metabolism associated with IR, one notable manifestation is the decreased levels of HDL cholesterol.

In the IR state, there is a notable decrease in HDL‐C levels, primarily attributed to a decrease in the HDL_2_ subtype, which is partly due to a shortage of an enzyme called lipoprotein lipase.^36^ Another study suggests that while high levels of cholesterol‐rich HDL_2_ are linked to lower serum triglyceride levels and IR, the presence of small, cholesterol‐poor HDL_3_ does not exhibit the same association.^37^ It was also found that patients with type 1 diabetes who were treated with insulin products had higher HDL_2_‐C than those with T2DM. Moreover, individuals with T2DM who were getting insulin treatment exhibited elevated levels of HDL_2_‐C compared to those patients who were not receiving insulin therapy.^38^ These findings indicated that the administration of exogenous insulin promotes an elevation in HDL_2_‐C levels, whereas IR mitigates the ability of insulin to raise HDL_2_‐C levels.^39^ It's noteworthy to mention that other factors besides HDL contribute to the correlation with IR; for instance, in Caucasians, the TG/HDL ratio is associated with IR.^40^ In men, a ratio of 3.5 or above, and in women, a ratio of 2.5 or above, suggest the existence of IR.^40^ Collectively, these insights highlight the intricate relationship between HDL and IR, emphasizing the importance of HDL subtypes and other factors in understanding and managing IR.

## 5 CAUSES OF LOW HDL‐C: PREDISPOSING FACTORS FOR DIABETES

**Figure 1:**
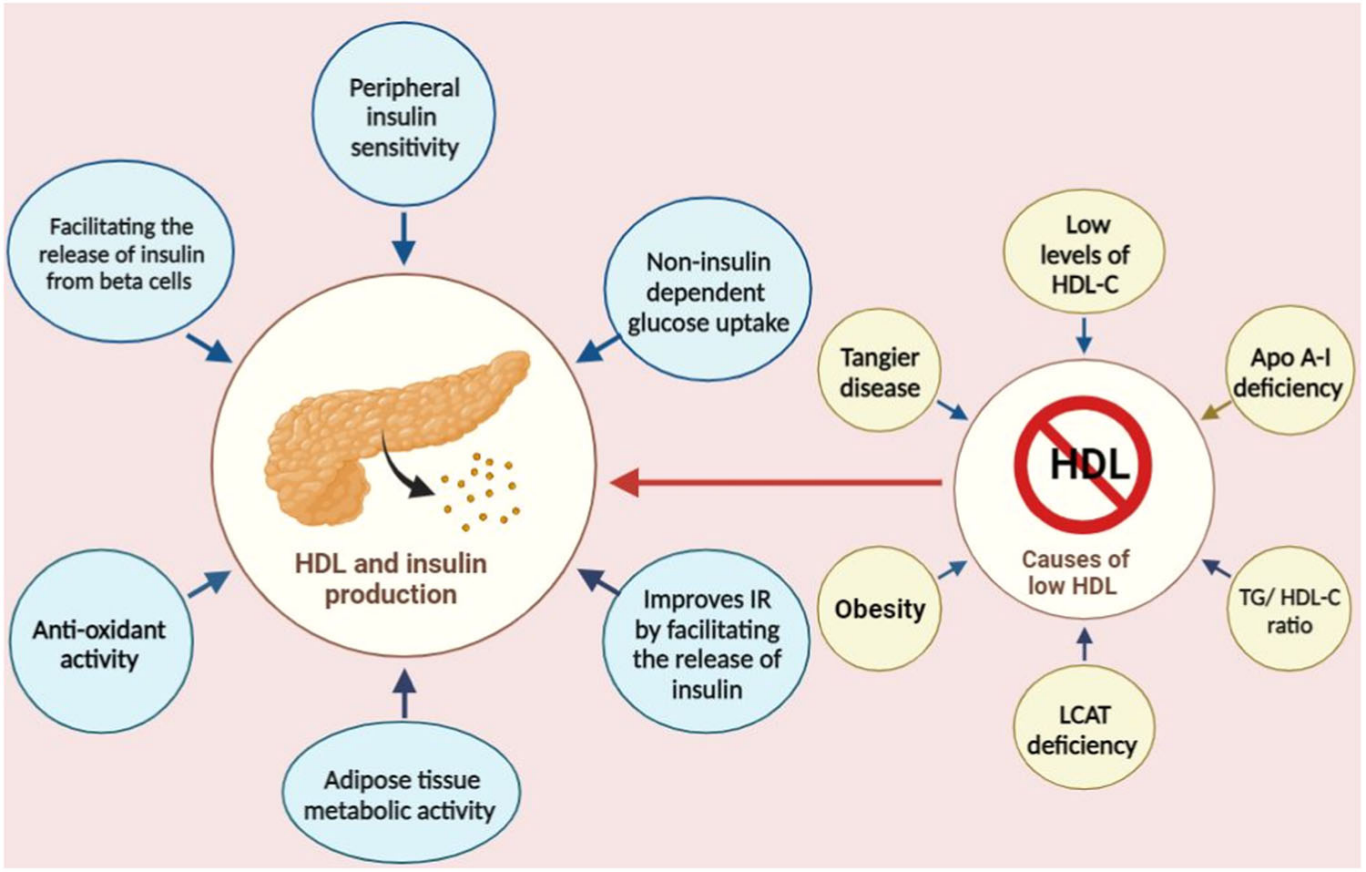
Different factors, such as hypoalphalipoproteinemia that manifests as a consequence of genetic factors, as well as secondary causes arising from lifestyle choices and underlying medical conditions that decrease the level of HDL, could be associated with DM. IR, insulin resistance; LCAT, lecithin‐cholesterol acyltransferase; TG, Triglyceride.

As mentioned in the previous sections, there is a notable decrease in HDL‐C levels in the IR state. To this end, other factors that decrease the level of HDL‐C (Figure 1) could be associated with diabetes, and in this part, causes of low HDL‐C will be discussed. Notably, low HDL‐C may be caused by either primary (familial) or secondary disorders.

### 5.1 Primary and familial disorders associated with low levels of HDL‐C

Low HDL levels in the blood are a sign of familial hypoalphalipoproteinemia. This is caused by mutations that turn off Apo A‐I, ABCA1, LCAT, secretion‐associated Ras‐related GTPase 1B (SAR1B), and ABCG1.^41^ In 1981, *Vergani* et al. described familial HDL deficiency as a familial aggregation of low HDL‐C (less than 33 mg/dL) and Apo A (about 50% of normal levels) in the presence of normal very low‐density lipoprotein (VLDL) and LDL cholesterol. In the affected members of this monogenic disease, no disorder that would alternatively lower the HDL levels, such as nephropathy, liver disease, overweight, or cigarette smoking, was present.^42^ In this concept, to define familial hypoalphalipoproteinemia, we need the following criteria: (1) low HDL‐C level with the presence of normal levels of VLDL and LDL‐C; (2) no evidence of diseases or any factors to which low HDL levels might be secondary; and (3) having an immediate family member with a similar lipoprotein pattern.^42^ According to studies done on different populations, 18.7% of individuals with very low HDL‐C levels carry rare genetic variants of great effect and 19.3% carry common low‐effect variants.^43^ Thus, the genetic basis of low HDL‐C levels is often regarded as polygenic.^44^

### 5.2 Apo A‐I deficiency

Apo A‐I is the main protein component of plasma HDL.^45^ Patients with Apo A‐I deficiency have an undetectable and thus complete loss of Apo A‐I in their plasma due to a lack of Apo A‐I production.^46^ A DNA inversion containing portions of the structural gene for Apo A‐I as well as the deletion of the complete Apo A‐I cluster on chromosome 11 are the basic defects that lead to Apo A‐I deficiency.^47^ A complete loss of Apo A‐I results in a profound decrease in HDL‐C.^48^, ^49^ Patients with an Apo A‐I defect have no Apo A‐I in their plasma and normal levels of LDL‐C and TGs.^50^ Heterozygous carriers have 50% of the HDL‐C and Apo A‐I levels of a normal individual and do not show any specific clinical symptoms.^51^, ^52^

### 5.3 Tangier disease

Tangier disease, also called familial alpha lipoprotein deficiency, is caused by a severe lack of HDL‐C in the plasma, which causes cholesteryl esters to build up in the body's tissues.^53^ This disorder is due to mutations in the gene that codes for ABCA1 and is inherited in an autosomal recessive manner.^54^, ^55^ ABCA1 helps the efflux of intracellular cholesterol from peripheral cells to lipid‐poor A1, the key first step of RCT.^56^ It's noteworthy to mention that such patients with Tangier disease are characterized by a profound deficiency of HDL‐C (<5 mg/dL) and Apo A‐I levels (4 mg/dL) and the presence of only the preβ−1 HDL fraction of HDL.^53^, ^57^ Fibroblasts in the mentioned patients showed defective cholesterol efflux to apolipoprotein. This phenomenon leads to Apo A‐I not being lapidated appropriately and thus being rapidly cleared by the kidney, which consequently decreases the levels of Apo A‐I and HDL.^58^, ^59^, ^60^ Since hepatic cells play a vital role in HDL homeostasis, the deletion of the intestinal ABCA1 gene leads to a 30% decrease in plasma HDL, and the deletion of both hepatic and intestinal ABCA1 results in a 90% decrease in plasma HDL levels.^61^

### 5.4 LCAT deficiency

LCAT is mostly attached to HDL and helps cholesterol become more stable by moving a fatty acid from phosphatidylcholine to cholesterol.^61^, ^62^, ^63^ LCAT is important for the maturation of HDL particles. LCAT deficiency is an autosomal recessive disorder that results from a mutation in the LCAT gene and is characterized by either a complete lack of the enzyme, known as familial LCAT deficiency (FLD), or a partial lack of the enzyme, known as fish‐eye disease (FED).^61^, ^62^, ^64^, ^65^ Subsequently, it was reported that there are two activities associated with LCAT: α‐LCAT activity, which affects both LDL and HDL, and β‐LCAT activity, which affects only HDL.^66^ In FLD, there is a deficiency in α‐LCAT activity, and LCAT is unable to esterify cholesterol in both HDL and LDL. On the other hand, β‐LCAT activity is affected in FED, and LCAT is unable to esterify cholesterol in HDL but is able to esterify cholesterol in LDL.^61^, ^62^, ^66^ In LCAT disorder, because cholesterol cannot be converted to cholesterol esters, free cholesterol levels are elevated in plasma and peripheral tissues. This process leads to a lack of mature HDL particles and thus a rapid clearance of Apo A‐I.^51^, ^58^ The lipid and lipoprotein profiles in both FLD and FED patients demonstrate low HDL‐C (<10 mg/dL) and Apo A‐I levels (<50 mg/dL), elevated TGs, and decreased LDL‐C levels.^50^

## 6 SECONDARY CAUSES ASSOCIATED WITH LOW LEVELS OF HDL‐C

There are many secondary conditions that are associated with hypoalphalipoproteinemia, including obesity, TG/HDL‐C ratio, sedentary lifestyle, cigarette smoking, hypertriglyceridemia, a very low‐fat diet, malabsorption, malnutrition, end‐stage renal disease, and severe inflammatory disease.^67^ To this end, in this section, some of the most important secondary causes of low levels of HDL‐C will be discussed.

### 6.1 TG/HDL‐C ratio

A previous study demonstrated that high levels of the TG/HDL‐C ratio are associated with obesity, metabolic syndrome, and IR.^68^ It is considered that high levels of the TG/HDL‐C ratio result in the accumulation of cholesterol as a result of reduced HDL‐C levels, which leads to β‐cell dysfunction.^69^, ^70^

Clinical evidence has demonstrated that higher levels of the TG/HDL‐C ratio can accelerate the progression of T2DM.^16^ It is also revealed that the correlation between this ratio and the incidence of DM is nonlinear.^71^ Increased TG to HDL‐C indicates an emerging risk factor for developing T2DM and is positively related to the prevalence of diabetes.^16^, ^71^ According to these findings, there is a positive, nonlinear relationship between the TG/HDL‐C ratio and diabetes; however, to establish this relationship among different ages and ethnic groups, further research should be undertaken.

### 6.2 Obesity

Over the past few decades, obesity prevalence has increased dramatically due to a change in lifestyle. Besides, the significant role of obesity in developing T2DM and its other complications, such as dyslipidemia and cardiovascular disease (CVD) risk, has been demonstrated.^72^ Obesity is a well‐established risk factor for T2DM, and it has been demonstrated that metabolically healthy obese adults have a higher incidence of DM compared with metabolically healthy normal‐weight adults.^73^ Furthermore, obesity is associated with the incidence of the metabolic syndrome, which includes related risk factors for diabetes and CVD. Researchers have also found that there is a relationship between metabolic syndrome and the incidence of abdominal obesity and IR.^74^

Abdominal obesity is often accompanied by abnormalities in plasma lipid levels, which have been observed in patients with metabolic syndrome too.^72^, ^74^ These alterations include increased TG levels, reduced HDL‐C, and structurally abnormal LDL‐C, which is called obesity dyslipidemia.^72^, ^74^, ^75^ The higher level of total cholesterol in HDL was associated with reduced T2DM risk, while higher cholesterol esters in large very‐low‐density lipoprotein (VLDL) particles were associated with increased T2DM risk.^76^ To this end, a decrease in HDL‐C levels in obese patients is an emerging risk factor for developing T2DM. To uncover the different aspects of the correlation between obesity, HDL‐C, and the development of DM, further studies should be conducted on different ethnicities.

## 7 THE MANAGEMENTS OF LOW HDL: THE POSSIBLE PREVENTION WAY FOR DIABETES

Treatments for low HDL‐C have received a lot of attention in recent years. A patient may be considered for the administration of therapeutic medications that raise HDL‐C levels if their TG levels are between 200 and 400 mg/dL and their HDL‐C levels are less than 40 mg/dL.^77^, ^78^ Combining lifestyle changes with medication, when necessary, can successfully increase HDL cholesterol levels and have an impact on T2DM management.

### 7.1 Lifestyle modifications

For patients with T2DM, therapeutic lifestyle changes are strongly recommended to regulate blood glucose levels.^79^ To achieve this, patients should initially recognize and address secondary factors as a primary step in their treatment. These factors include smoking cessation, a low‐fat diet, weight control, increased physical activity, avoiding substances that are known to reduce HDL levels, and having optimal diabetes control.^78^ In this regard, it has been evident that smoking is related to HDL‐C levels and causes them to fall to a minor extent, but there is not enough hard evidence to show that its correction will normalize the low content of HDL in the blood. Some studies suggest that smoking cessation may raise HDL‐C levels in thirty days by up to 5%.^79^, ^80^, ^81^, ^82^, ^83^

Additionally, as mentioned earlier, there is an inverse relationship between obesity and HDL‐C levels. Adopting a healthy diet and losing weight will help increase lipoprotein levels and thus help maintain optimal HDL‐C levels.^81^ Low HDL levels are associated with a low fat diet. In contrast, it is not recommended to increase a patient's dietary fat for the sole purpose of increasing their HDL levels. Studies suggest that dietary management should follow the National Cholesterol Education Program guidelines.^78^ Finally, there's no denying that physical activity is critical to achieving optimal health. It serves as a crucial element in leading a healthy lifestyle, offering numerous advantages beyond merely keeping a healthy weight, BMI, and improving muscular endurance. Noteworthy, it has been found that long‐distance runners have significantly higher HDL levels than those with sedentary lives.^84^, ^85^ It has been demonstrated that regular exercise can increase HDL‐C in plasma.^86^, ^87^, ^88^, ^89^

### 7.2 Therapeutic agents

Statins, also known as HMG‐COA reductase inhibitors, treat diabetic dyslipidemia by inhibiting the 3‐hydroxy‐3‐methylglutaryl coenzyme A (HMG‐COA) reductase enzyme.^25^ Noteworthy, atorvastatin and pravastatin are two of the most important drugs in the statin family (Table 1).^78^, ^90^ These drugs facilitate the removal of LDL by upregulating LDL receptors and almost doubling HDL‐C levels by 15%.^91^, ^92^ Statins also improve HDL's ability to efflux cholesterol.^99^ A recently published study reported that statins have limited effectiveness in reducing plasma TG levels.^90^ As a result, statins raise HDL levels, reduce LDL, and cause a mild reduction in plasma TG levels.^25^

**Table 1:** Therapeutic agents for managing HDL level and prevention of diabetes.

Drugs	Mechanism	Members	The effect on HDL	The effects on other lipids	Glucose tolerance	Side effects	References
**Statins**	Facilitating the LDL removal by upregulating LDL receptors	Atorvastatin	15% increase	LDL and TG reduction	Increased IR and insulin secretion	Neuromuscular side effects	[78, 90, 91, 92, 93, 94]
		Pravastatin					
**Niacin**	Inhibiting the production of LDL‐C precursors	Niacor	15%‐35% increase	LDL reduction	Minimally worsened	Limited use due to difficulty in tolerating clinically relevant dose	[10, 88, 92, 95, 96]
		Niaspan					
**Fibrates**	Inducing the expression of LpL, inhibition of the Apo C‐III expression, and Apo B‐100 and VLDL synthesis	Gemfibrozil	10%‐15% increase	LDL, TG, and VLDL reduction	Gemfibrozil is more efficient in T2DM patients	Increased serum creatinine and kidney disease	[92, 95, 97, 98]
		Bezafibrate					
**CETP inhibitors**	Inhibiting the transfer of cholesterol esters from HDL‐C to LDL or IDL in exchange for TG	Torcetrapib	106% increase (dose‐dependent)	LDL reduction	A combination of CETP inhibitors and infusion of rHDL (Apo A‐I and phospholipids) is beneficial for T2DM patients	CETP inhibitors are required for LDL uptake by RCT^a^ pathway	[12, 90, 95, 99, 100, 101]
		Dalcetrapib (JTT‐705)	34% increase (dose‐dependent)				

Abbreviations: Apo, apo lipoprotein; CETP, cholesterol ester transfer protein; HDL, high‐density lipoprotein; HDL‐C, high‐density lipoprotein cholesterol; IDL, intermediate‐density lipoprotein; IR, insulin resistance; LDL, low‐density lipoprotein; LDL‐C, low‐density lipoprotein cholesterol; LPL, lipoprotein lipase; RCT, reverse cholesterol transport; rHDL, reconstituted high‐density lipoprotein; T2DM, type 2 diabetes mellitus; TG, triglyceride; VLDL, very low‐density lipoprotein.

^a^ RCT pathway; is a critical pathway in the human body and inhibiting this pathway could cause irreversible damages.

Furthermore, niacin, nicotinic acid, or vitamin B_3_, is a water‐soluble vitamin that lowers LDL‐C and raises HDL‐C.^102^ In recent studies, niacin has been found to be the most effective HDL raiser, with niacin increasing HDL‐C by 15%–35%.^2^, ^10^, ^95^, ^96^ Niacin inhibits the production of LDL‐C precursors and also reduces the uptake of HDL‐C, thereby increasing the level of HDL‐C in the peripheral tissues.^95^ Niacin increases HDL‐C levels by improving cholesterol efflux and inhibiting TG release from adipose tissue.^103^ Niacin also improves HDL's anti‐inflammatory and antioxidant functions in patients with diabetes.^99^

Fibrates, peroxisome proliferator‐activated receptor alpha (PPARα) agonists, reduce TG and LDL levels, while these compounds raise the level of HDL.^10^, ^95^, ^104^, ^105^ Fibrates include gemfibrozil, fenofibrates, and bezafibrate.^97^ In recent studies, fibrates have been shown to reduce TG by up to 50% by reducing VLDL synthesis.^99^, ^104^ Furthermore, these drugs increase HDL levels by 10%–15% by increasing transcription of the human Apo A‐I gene.^92^, ^95^ A study demonstrated the benefits of gemfibrozil in patients with T2DM.^92^ There is also evidence that a combination therapy with statins and fibrates may be more effective in treating diabetic patients.^92^

In addition to the mentioned drugs, cholesterol ester transfer protein (CETP) inhibitors are also one of the first drugs to increase HDL‐C levels.^103^ CETP is a plasma glycoprotein that facilitates the transfer of cholesteryl esters from HDL‐C to lipoproteins containing Apo‐B like LDL and VLDL in exchange for TG.^92^, ^95^, ^106^ As a result of inhibiting this step, CETP inhibitors increase HDL‐C levels.^92^ However, different adverse effects were reported for these drugs.^12^ CETP is also required for cholesterol uptake from LDL through the RCT pathway.^100^ Dalcetrapib (JTT‐705) was the first functional CETP inhibitor.^101^ This agent caused a 34% increase in HDL‐C.^92^ Another agent is Torcetrapib, which results in a dose‐dependent increase in HDL‐C levels up to 106% and a reduction in LDL‐C levels up to 42%.^107^ Notably, it was shown that administering a combination of CETP inhibitors with an infusion of rHDL increased HDL levels in patients with T2DM.^90^, ^99^ Collectively, some drugs (Table 1) could enhance the level of HDL and reduce the chance of T2DM; however, the exact role of these drugs in the reduction of diabetes should be evaluated in future studies.

## 8 HDL AND DIABETIC COMPLICATIONS

### 8.1 Cardiovascular disease

Numerous studies have demonstrated that HDL‐C is strongly and inversely related to CVD.^108^ HDL‐C inhibits atherogenic events through several mechanisms. For instance, HDL elevates cholesterol efflux, which promotes RCT.^12^ RCT is one of the most significant cardioprotective mechanisms and leads to cholesterol efflux from cells to HDL.^109^ This cholesterol is transported by HDL to the liver for processing before being excreted in bile and feces.^109^ Patients with diabetes are more likely to develop CVDs than those without diabetes.^110^ The accelerated development of atherosclerotic plaque in T2DM is one of the major causes of coronary artery disease and could lead to the death of patients.^25^, ^111^

It's noteworthy to mention that T2DM patients are at an increased risk of stroke.^112^ In recent studies, attempts to elevate HDL levels to decrease the risk of CVD have been disappointing.^12^ This suggests that some factors beyond HDL's concentration are responsible for the incidence of CVD.^12^ The loss of HDL's antiatherogenic function is called HDL dysfunction.^103^ HDL in healthy individuals with normal lipid and glucose levels, inhibits lipid oxidation.^113^ DM leads to a reduction in HDL's antioxidant capacity due to hyperglycemia and glycation.^108^ Accordingly, HDL is less cardioprotective in patients with DM, and diabetic patients are more likely to develop CVD; however, more confirmatory studies are needed in this field.^110^, ^113^

### 8.2 Kidney disease

The presence of diabetes leads to several changes in the composition of the glomerulus membrane, including glycosylation, which causes endothelial dysfunction.^114^ Nearly 20%–40% of patients with diabetes are at risk of developing diabetic nephropathy, a serious complication of DM.^115^, ^116^ Hyperglycemia and arterial hypertension are significant in diabetic nephropathy. There is evidence that variations in the lipid profile, such as increased TG and reduced HDL levels, contribute to diabetic nephropathy.^115^, ^117^ The accumulation of lipids in the glomerulus has been linked to glomerular damage in recent studies.^117^

A Recently published study reported that the prevalence of diabetic kidney disease (DKD) has been linked to high levels of TG and low levels of HDL.^117^ As mentioned previously, reduced levels of HDL‐C in plasma are associated with DM.^118^ DKD reduces HDL's ability to remove cholesterol, and it is considered that this dysfunction is due to Apo A‐IV and Apo D.^118^ Increased levels of Apo A‐IV and Apo D could lead to the chemical alteration of HDL, and this alteration could lead to HDL dysfunction.^118^ To this end, HDL has been demonstrated to be chemically altered and dysfunctional in patients with diabetic kidney disease; however, there is a need for further research on this relationship to uncover novel perspectives.

### 8.3 Nonalcoholic fatty liver disease (NAFLD)

DM is a risk factor for NAFLD. NAFLD is the most common form of liver disease, and it is related to the incidence of DM.^119^, ^120^ The liver plays a crucial role in regulating glucose and lipoprotein metabolism.^121^, ^122^ The presence of NAFLD is also associated with an increase in TG levels and a reduction in HDL levels.^122^ HDL concentrations can vary in NAFLD patients.^121^ As a consequence of liver fatty disease, HDL is also functionally impaired and altered.^123^ Therefore, HDL levels are low and often dysfunctional in NAFLD, and further studies should be conducted to clarify the exact relationship between HDL and the prevalence of NAFLD.

## 9 CONCLUSION

The intricate interplay between HDL and diabetes reveals the fundamental role of HDL‐C in glucose metabolism, IR, and the development of diabetic complications. Recent scientific advancements have made exciting breakthroughs in understanding the benefits of HDL‐C, revealing its significance beyond atheroprotection, and placing HDL back in the research spotlight. To this end, more recently, HDL's antidiabetic function and its vital role in maintaining optimal glucose homeostasis and promoting insulin sensitivity have become the focus of research. HDL plays a significant role in glycemic control and insulin secretion. In several studies, T2DM and HDL‐C have been demonstrated to have mutual effects. Additionally, T2DM has been shown to impair HDL's function, including impaired cholesterol efflux capacity or impaired endothelial repair due to changes in HDL's composition. Moreover, lifestyle modifications encompassing regular exercise, a balanced diet, and weight management play a pivotal role in optimizing HDL levels. Furthermore, therapeutic agents such as exogenous HDL or Apo A‐I exhibit promising potential for increasing HDL‐C levels, presenting viable options for intervention in individuals with diabetes. By emphasizing the significance of HDL‐C in glucose regulation, exploring causes of hypoalphalipoproteinemia, promoting lifestyle modifications, and considering therapeutic interventions, we can strive to mitigate the burden of diabetic complications. As mentioned earlier, elevated levels of HDL‐C are more protective in uncomplicated T2DM. Collectively, the HDL can be considered a potential therapeutic target for T2DM. However, the exact interactions between HDL and diabetes have not been elucidated yet; therefore, continued research efforts hold great promise for advancing our understanding and optimizing outcomes for individuals living with diabetes.

## AUTHOR CONTRIBUTIONS

**Ali Bayat Bodaghi**: Conceptualization; data curation. **Erfan Ebadi**: Software; writing—original draft. **Mohammad Javad Gholami**: Writing—original draft. **Reza Azizi**: Writing—review and editing. **Aref Shariati**: Writing—original draft; writing—review and editing.

## CONFLICT OF INTEREST STATEMENT

The authors declare that the research was conducted in the absence of any commercial or financial relationships that could be construed as a potential conflict of interest.

## ETHICS STATEMENT

The authors have nothing to report.

## TRANSPARENCY STATEMENT

The lead author Aref Shariati affirms that this manuscript is an honest, accurate, and transparent account of the study being reported; that no important aspects of the study have been omitted; and that any discrepancies from the study as planned (and, if relevant, registered) have been explained.

## Data Availability

The authors confirm that the data supporting the findings of this study is available within the article.
